# First-in-human left atrial appendage closure using the WATCHMAN FLX Pro device: a case report

**DOI:** 10.1093/ehjcr/ytae135

**Published:** 2024-03-19

**Authors:** Jens Erik Nielsen-Kudsk, Anders Kramer, Asger Andersen, Won Yong Kim, Kasper Korsholm

**Affiliations:** Department of Cardiology, Aarhus University Hospital, Palle Juul-Jensens Boulevard 99, Aarhus N 8200, Denmark; Department of Cardiology, Aarhus University Hospital, Palle Juul-Jensens Boulevard 99, Aarhus N 8200, Denmark; Department of Cardiology, Aarhus University Hospital, Palle Juul-Jensens Boulevard 99, Aarhus N 8200, Denmark; Department of Cardiology, Aarhus University Hospital, Palle Juul-Jensens Boulevard 99, Aarhus N 8200, Denmark; Department of Cardiology, Aarhus University Hospital, Palle Juul-Jensens Boulevard 99, Aarhus N 8200, Denmark

**Keywords:** Left atrial appendage, Left atrial appendage closure, Left atrial appendage occlusion, Case report

## Abstract

**Background:**

Device-related thrombosis (DRT) is a known complication to left atrial appendage closure (LAAC). The surface of a LAAC device should ideally have antithrombotic properties. The novel WATCHMAN FLX Pro (WFP) incorporates a fluoropolymer-coated fabric membrane designed to increase thromboresistance and facilitate endothelialization. Such features could potentially allow for a minimal post-procedural antithrombotic regimen. Radiopaque platinum markers at the device shoulders and a large 40 mm device are other novel features of the WFP.

**Case summary:**

A 75-year-old man with atrial fibrillation was referred for LAAC due to prior subdural haemorrhage during direct-acting anticoagulation treatment. He underwent the first-in-human WFP implantation as part of the WATCHMAN FLX Pro CT study (NCT05567172). Computed tomography (CT) was used for pre-planning, and the procedure was performed under local analgesia guided by intracardiac echocardiography from the left atrium (LA) without any complications. Post-procedural antithrombotic treatment consisted of acetylsalicylic acid 75 mg/day only, and 45-day CT, transoesophageal echocardiography (TEE), and magnetic resonance imaging demonstrated optimal device position with complete LAAC. Hypoattenuated thickening (6 mm) appeared on the device as a smooth surface in continuity with the left atrial wall on CT and TEE. A specific magnetic resonance T_1_-weighted scan, used for visualization of fresh thrombus, suggested this to represent tissue ingrowth rather than thrombus.

**Discussion:**

The advanced follow-up imaging protocol suggested a good WFP implantation result with signs of tissue ingrowth at 45 days. The added radiopaque markers facilitated optimal deployment, evaluation of device stability during tug test, and assessment of device protrusion into the LA.

**Learning points**

- The WATCHMAN FLX Pro features an added fluoropolymer coating of the fabric membrane for increased thromboresistance and facilitation of endothelium coverage. Three radiopaque platinum markers at the device shoulders increase fluoroscopic visibility, and device size range now includes a 40 mm device.
- Antithrombotic properties built into the surface of a LAAC device potentially allow for a minimal post-procedural antithrombotic regimen.
- Post-procedural antithrombotic therapy was limited to single antiplatelet therapy, and 45 days imaging with CT, TEE, and MRI documented an optimal result with signs of tissue ingrowth.

## Introduction

The left atrial appendage (LAA) is the principal source of thromboembolism in atrial fibrillation (AF).^1^ Percutaneous LAA closure (LAAC) can prevent systemic thromboembolism in AF patients. However, device-related thrombosis (DRT) occurs in 1–4% of LAAC cases involving a risk of thromboembolic complications.^2–4^ The gradual endothelialization of the device taking place over months after implantation is supposed to protect against DRT. However, DRT may still develop late (even years) after LAAC even despite complete sealing of the LAA.^5,6^

**Figure 1:**
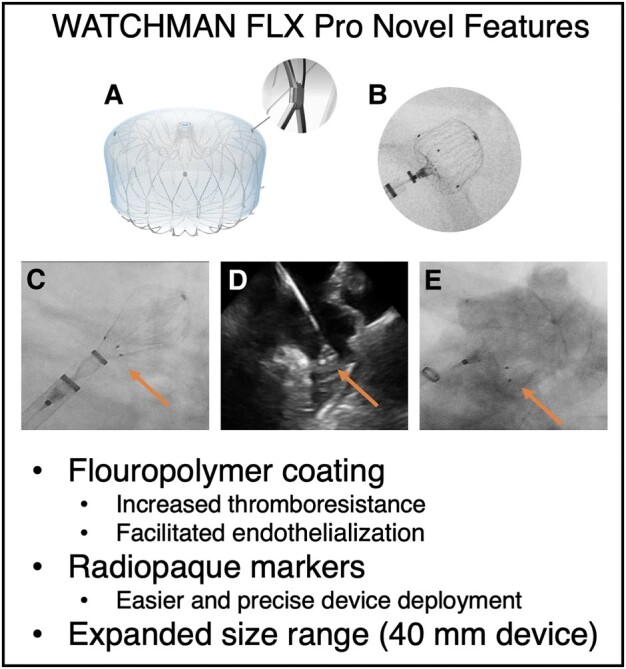
(*A*) WATCHMAN FLX Pro device with magnified radiopaque markers. (*B*) Fluoroscopic image showing aligned markers at the shoulders of the device. (*C*) Device in ball configuration during implantation. (*D*) Markers seen on intracardiac echocardiography. (*E*) Device deployed with aligned markers at the level of the left atrial appendage ostium. The listed features are new in the WATCHMAN FLX Pro compared with the WATCHMAN FLX. Arrows points at the radiopaque markers.

Antithrombotic treatment is needed in the post-LAAC period to prevent DRT. The most frequently used regimen in Europe is dual antiplatelet therapy (DAPT) with clopidogrel and acetylsalicylic acid (ASA) for 1–6 months followed by ASA alone.^2^ However, LAAC patients are at high risk of bleeding and a minimalistic post-LAAC antithrombotic treatment is desirable to avoid adverse bleeding. The novel WATCHMAN FLX Pro (WFP; Boston Scientific, Marlborough, USA) incorporates a new fluoropolymer coating (polyvinylidene fluoride-co-hexafluoropropylene) on the fabric membrane covering the device to increase thromboresistance and facilitate rapid tissue ingrowth across the device surface (*Figure 1*). These features of the new coating were demonstrated in pre-clinical animal experiments^7^ and may potentially allow single antiplatelet therapy to be sufficient as post-procedural treatment for this novel device. This will need confirmation in a dedicated clinical trial.

Additionally, WFP is equipped with three radiopaque platinum markers at the proximal and widest part of the device (device shoulders), thereby increasing its fluoroscopic visibility (*Figure 1A* and *B*). These markers may facilitate positioning and deployment of the device at the desired landing zone. Moreover, the range of device sizes has been expanded with a 40 mm device for closure of very large LAAs. The purpose of this case report was to present the first-in-human LAAC with the novel WFP device.

## Case presentation

A 75-year-old male with AF and hypertension was referred for LAAC due to subdural haemorrhage (SDH). He had two prior ablation procedures for AF and previous neurosurgery with removal of a meningioma over one of the brain hemispheres. The SDH occurred 4 months after neurosurgery while being treated with direct-acting oral anticoagulation (DOAC). He was considered unsuitable for long-term DOAC treatment. His CHA_2_DS_2_VASc and HAS-BLED scores were 3. Echocardiography was normal except for trivial mitral and aortic regurgitation. An electrocardiogram (ECG) indicated sinus rhythm, and physical examination was unremarkable. Metoprolol and amlodipine were given as antihypertensive treatment, and there were no other concomitant medical conditions.

**Figure 2:**
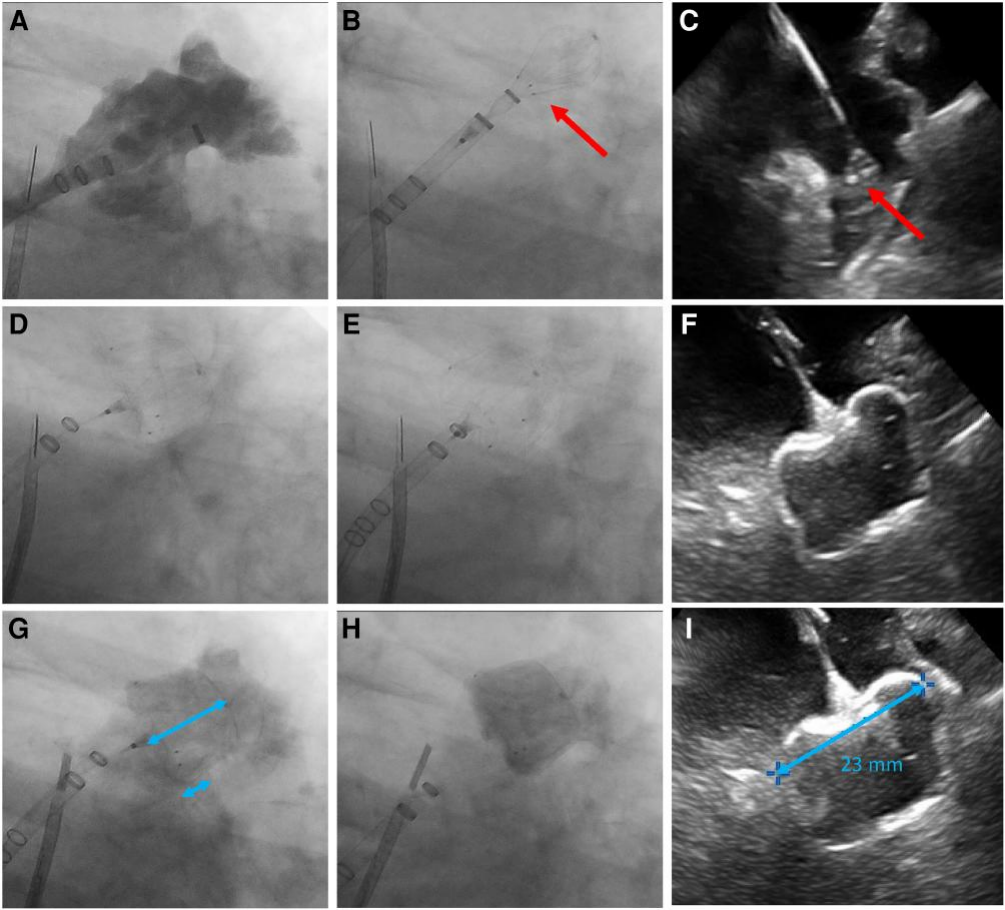
Fluoroscopic and intracardiac echocardiography images showing left atrial appendage closure with the WATCHMAN FLX Pro device. (*A*) Selective left atrial appendage angiogram. (*B* and *C*) Device in ball shape and arrow points to the three radiopaque markers. (*D*) Device fully deployed. (*E*) Tug test performed by pulling in the device with the delivery cable. (*F*) Intracardiac echocardiography image showing the fully deployed device. (*G*) Left atrial appendage angiogram with the three markers aligned in a straight line. The blue bars show the length of the small infero-posterior shoulder and the full length of the device. (*H*) Device released from the core wire. (*I*) Intracardiac echocardiography image showing a shoulder-to-shoulder measurement to estimate device compression.

The patient underwent the first-in-human implantation of a WFP device as part of the WATCHMAN FLX Pro CT study (NCT05567172). Pre-procedural planning was done using cardiac computed tomography (CT) showing a chicken wing LAA with a landing zone of 24 × 18 mm and a depth of 17 mm (*Figure 2*; Supplementary material online, Video  *S1*) without any signs of LAA thrombus. He was planned for a 27 mm WFP device.

The procedure was performed under local analgesia guided by intracardiac echocardiography (ICE). The procedural workflow has previously been described in detail.^8^ In brief, ICE from the right atrium guided an infero-posterior puncture of the interatrial septum. With a supportive guidewire in the left upper pulmonary vein, a double-curve 14F access sheath was advanced over the wire to increase the puncture hole in the septum followed by advancement of the ICE catheter along the guidewire into the left atrium (LA). The access sheath with a pigtail catheter in front was advanced into the LAA at the level of the device landing zone followed by selective LAA angiography (*Figure 2A*; Supplementary material online, Video S1). After deairing the WFP device, the catheter with the device inside was advanced inside the access sheath to the landing zone. The 27 mm WFP was deployed to the ball configuration (*Figure 2B*) and positioned using the radiopaque markers at the level of the landing zone. The platinum markers were clearly visible on fluoroscopy and ICE (*Figure 2C*). After co-axial alignment of the device and LAA, and positioning at an optimal depth, the device was fully deployed by further back sheathing the delivery sheath (*Figure 2D* and *F*). Device stability was tested by gently tugging the core wire while observing device movements (*Figure 2E*). The radiopaque markers clearly demonstrated that the device returned to its position after tugging, confirming no device displacement or instability. There was a small protrusion of the device into the LA at the infero-posterior shoulder. The degree of shoulder protrusion could be readily quantified by adjusting the C-arm position to get the three radiopaque markers in a straight line while performing a contrast angiogram (*Figure 2G*). Device compression was measured by ICE (23/27 mm; 15%; *Figure 2I*). The device was released after confirming the position, anchoring, size, seal (PASS) criteria (*Figure 2G* and *H*).

**Figure 3:**
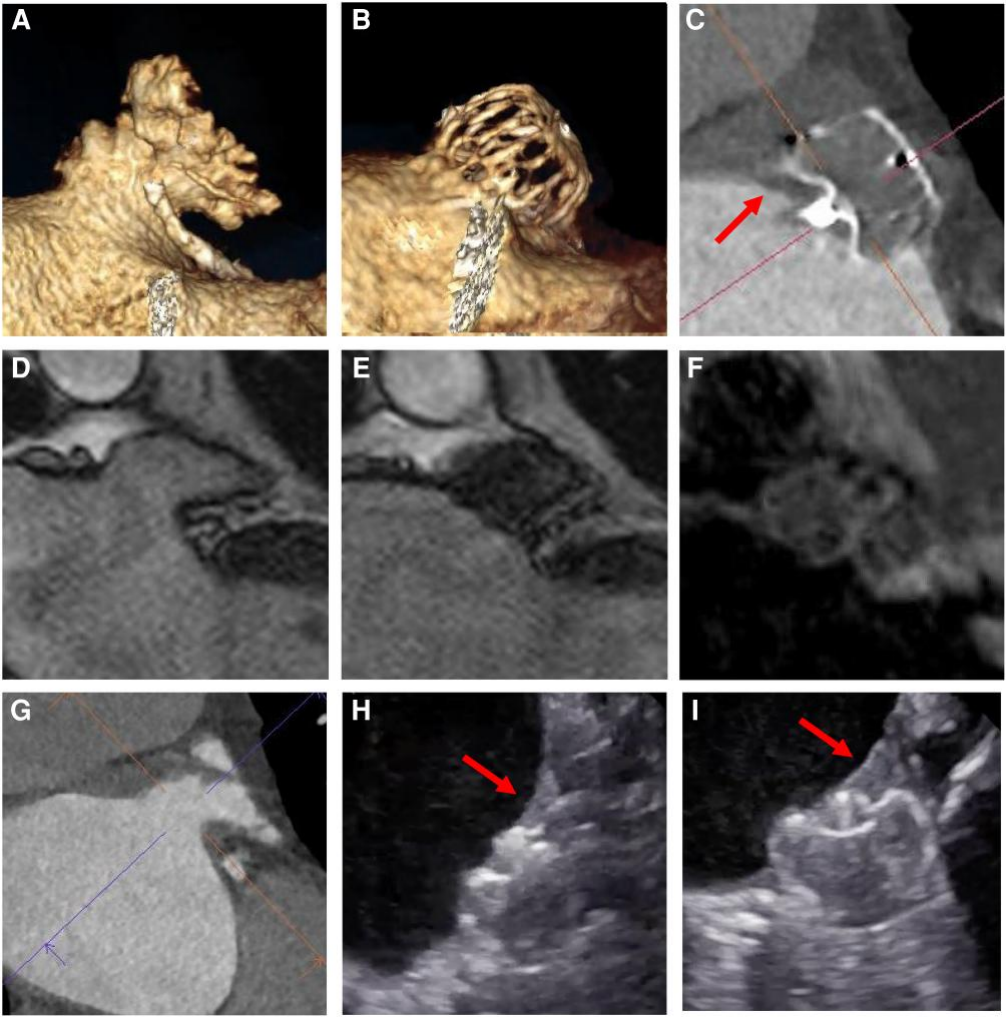
Cardiac computed tomography, transoesophageal echocardiography, and magnetic resonance imaging images obtained before left atrial appendage closure and at Day 45 after implantation of the first-in-human WATCHMAN FLX Pro device. (*A* and *G*) Computed tomography images of the left atrial appendage before closure showing a chicken wing anatomy. (*B* and *C*) Computed tomography images of the device implanted in the left atrial appendage showing complete closure. (*C*) Computed tomography showing hypoattenuated thickening at the upper half of the device with a smooth surface in continuation with the left atrial wall (arrow). (*D* and *E*) Magnetic resonance images showing the left atrial appendage before and after closure. (*F*) Specific bright-blood and black-blood phase sensitive magnetic resonance scan without any signs of fresh thrombus in the region of the hypoattenuated thickening. (*H* and *I*) Transoesophageal echocardiography images showing a layering on the upper half of the device with smooth surface and continuation with the left atrial wall (arrows).

Per the WATCHMAN FLX Pro CT protocol, the post-procedural antithrombotic treatment was ASA 75 mg/day alone, and follow-up was done by CT, transoesophageal echocardiography (TEE), and magnetic resonance imaging (MRI) at 14 days, 45 days, and 3 months. At 45 days, cardiac CT, TEE, and MRI concordantly showed the device in an optimal position with complete sealing of the LAA (Supplementary material online, Video S2). There was no pericardial effusion or peridevice leak (*Figure 3*). Computed tomography showed a ∼6 mm hypoattenuated thickening (HAT) at the superior half of the device as a smooth surface in continuity with the left atrial wall (*Figure 3C*).^9^ This was also seen on TEE (*Figure 3H* and *I*; Supplementary material online, Video S3). A specific 3D T_1_-weighted MR sequence was applied for visualization of fresh thrombus. This bright-blood and black-blood phase sensitive (BOOST) scan will light up with bright white in areas with fresh thrombus,^10^ but there was no such signal in the area of HAT, suggesting tissue ingrowth rather than thrombus. The patient is awaiting his 3-month imaging follow-up and has not had any adverse events including thromboembolism.

## Discussion

This is the first-in-human LAAC with implantation of the WFP device. It was pre-planned by cardiac CT and performed under local analgesia guided by ICE from the LA without complications. The markers increased the fluoroscopic visibility of the device facilitating precise deployment at the intended landing zone. On fluoroscopy, the distal hub marks the most distal part of the device, and the three new markers define the proximal and widest part of the device (device shoulders) that should be landed at the level of the circumflex artery perpendicular to the axis of the LAA. When a tug test is performed to assess mechanical stability, it can be difficult to determine if the device changes position. The three markers returning to a pre-tug position gives confidence in mechanical stability. The markers further helped to estimate device shoulder protrusion by aligning the C-arm projection to obtain a view of the three markers at a straight line perpendicular to the device. These device changes provided substantial information for the implanter in deployment and evaluation of device position inside the LAA.

In pre-clinical canine experiments with no post-procedural antithrombotic treatment, the novel WFP exhibited less DRT and tissue inflammation than Watchman FLX (WF) at 45 days.^7^  *In vitro* studies showed that WFP increased albumin adsorption compared with WF.^7^ Since albumin does not bind platelets, the preferential adsorption of albumin over other plasma proteins like fibrinogen caused less platelet adhesion and activation.^7^

In this first-in-human WFP implantation, cardiac CT and TEE showed HAT with a thickness of ≤6 mm covering the upper half of the device but exhibiting a smooth surface and continuation with the LA wall, suggesting this represents benign device healing.^9^ The BOOST magnetic resonance scan did not show signs of fresh thrombus. We believe that the observed HAT represents tissue overgrowth of a substantial portion of the device at 45 days. The patient received single antiplatelet therapy as the post-procedural medication regimen and did not experience any embolic events. This early report of a first-in-human LAAC with the novel WFP seems promising and in line with the design goals of low thrombogenicity and fast tissue ingrowth. Future imaging and clinical studies are needed to prove a potential benefit of the novel WFP in reducing the risk of DRT.

## Supplementary Material

ytae135_Supplementary_Data

## Data Availability

The data underlying this article are available in the article and in its online supplementary material.

## Lead author biography

Jens Erik Nielsen-Kudsk, MD, DMSc, is a clinical professor and head of structural heart interventions at Aarhus University Hospital in Denmark. He has carried out significant research related to transcatheter closure of the left atrial appendage, and he is one of the pioneers in using cardiac CT and intracardiac echocardiography for LAAC. Aarhus University Hospital, Denmark, is a large centre in Europe for LAAC.
